# Automated Ultrasound‐Based Analysis of Urethral Kinematics in Stress Urinary Incontinence: A Pilot Study

**DOI:** 10.1002/nau.70231

**Published:** 2026-02-09

**Authors:** Kourosh Kalayeh, J. Brian Fowlkes, Stephanie Daignault‐Newton, Payton Schmidt, James A. Ashton‐Miller, John O. DeLancey

**Affiliations:** ^1^ Department of Radiology University of Michigan Ann Arbor Michigan USA; ^2^ Department of Urology University of Michigan Ann Arbor Michigan USA; ^3^ Department of Biomedical Engineering University of Michigan Ann Arbor Michigan USA; ^4^ Department of Obstetrics and Gynecology University of Michigan Ann Arbor Michigan USA; ^5^ Department of Mechanical Engineering University of Michigan Ann Arbor Michigan USA

**Keywords:** computer vision, female pelvic floor, image processing, stress urinary incontinence, transperineal ultrasound, urethra, urethral support system

## Abstract

**Objectives:**

Stress urinary incontinence (SUI) has been linked to excessive urethral mobility, yet clinical evaluation has been largely limited to assessing maximal excursion rather than capturing the full dynamics of visible urethral movement. In this study, we hypothesize that an automated, ultrasound‐based method can objectively differentiate urethral mobility patterns between women with SUI and continent controls.

**Methods:**

We used a previously validated optical flow‐based algorithm to automatically track urethral motion from transperineal ultrasound images during cough, Valsalva maneuver, and pelvic muscle contraction (PMC) in 11 women with SUI and 10 continent controls. Urethral motion was assessed by defining three regions of interest along the urethra (proximal, mid, and distal). Segmental urethral kinematics were computed and statistically compared between groups.

**Results:**

Substantial variability and overlap between groups were observed, with coefficient of variation ranging 25%–90%. On average, women with SUI demonstrated significantly larger urethral displacement compared to controls, particularly at the proximal segment during Valsalva (10.6 ± 1.2 mm vs. 6.0 ± 0.6 mm, *p* < 0.01), with pronounced inferior‐posterior motion. Additionally, displacement between the upper and lower urethra was significantly larger in the SUI group (0.47 ± 0.10 mm*/*mm vs. 0.13 ± 0.03 mm*/*mm, *p* < 0.05), indicating localized hypermobility particularly near the proximal urethra. Maneuver‐specific differences were also noted within the SUI group, with Valsalva producing significantly larger and less uniform urethral movements compared to cough (10.6 ± 1.2 mm vs. 6.6 ± 0.5 mm, *p* < 0.05).

**Conclusion:**

Our results demonstrate that the automated method is capable of capturing urethral mobility characteristics associated with SUI. Significant inter‐individual variability in both continent and SUI groups indicates that urethral kinematics are heterogeneous. The detailed kinematic data have the potential to identify distinct sub‐types of urethral mobility, facilitating systematic comparisons with underlying structural and neuromuscular defects. This approach can move clinical evaluation from simple group comparisons toward personalized SUI diagnosis and targeted treatment selection. Future studies with larger sample sizes and inclusion of additional pelvic floor conditions will be needed to validate these findings and advance their translation into clinical practice.

## 1 Introduction

**FIGURE 1:**
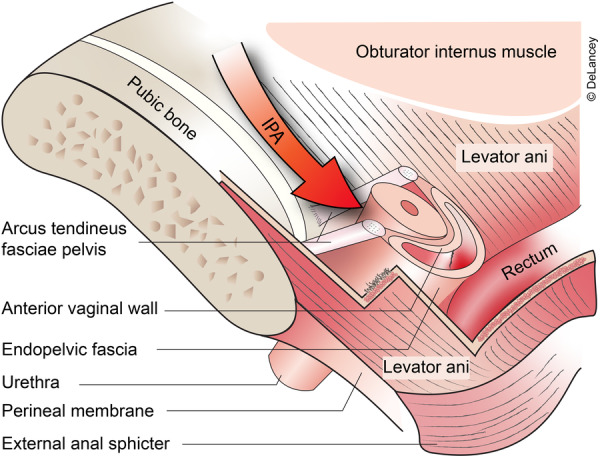
Urethral support structure.

Urethral support is an important aspect of stress urinary continence (SUI). The urethral support system is composed of connective tissue (endopelvic fascia) and striated muscle (levator ani muscles), which together form a supportive hammock beneath the urethra and bladder neck, under complex neuromuscular control from the central nervous system (CNS) [1]. The levator ani muscles attach to the supports at the junction of the upper and mid urethra, while distally the urethra is more fixed at the perineal membrane [2]. The endopelvic fascia attaches laterally at the arcus tendineus fascia pelvis proximally, see Figure 1. Failure or weakening in each of these components alone or in combination can disrupt urethral support, leading to variations in urethral motion. Studying these patterns using kinematics — the description of motion in terms of magnitude, direction and rotation—can provide deeper insights into urethral support.

Our understanding of how these factors influence urethral support and SUI is incomplete. More information about different failure modes and their effect on urethral function during stressor events could lead to better treatment selection tailored to the individual woman's underlying issue. For example, if the timing of the muscle contraction is faulty, training to pre‐contract the muscle may be effective, while if the muscle itself is disrupted, it may not. If the muscle is weak, strengthening exercises could help. If the connective tissue is too compliant, interventions aimed at stiffening the tissue might be beneficial.

Current urethral support assessments typically focus on maximal urethral excursion, neglecting the dynamic motion occurring during cough, Valsalva, or pelvic muscle contraction (PMC). An automated technique that captures the full dynamic richness of urethral displacement during stressor events could provide more comprehensive information than measuring only total excursion of the vesical neck. Although such dynamic methods have been described previously [3, 4], their cumbersome analytical processes have prevented wider adoption. Recently, we developed an automated computer vision‐based method that efficiently tracks movement on mid‐sagittal transperineal ultrasound (TPUS) images of any format across multiple segments of the pelvic floor as desired, capturing its rich dynamic movements [5]. The purpose of this study is to demonstrate the feasibility of quantifying detailed urethral kinematics in both continent and incontinent women. Additionally, we explore variability within groups to identify distinct individual failure modes, laying the groundwork for future mechanistic phenotyping and personalized SUI management. It is worth noting that, given the modest sample size of this study (21 subjects) and its exploratory design, the present work is best regarded as hypothesis‐generating, intended to demonstrate methodological feasibility and to motivate larger studies needed for validation and generalizability. The rich, quantitative data obtained by this method also opens possibilities for future integration with artificial intelligence and machine learning techniques to identify key patterns and parameters that may not be readily apparent with traditional analysis.

## 2 Methods

We used an automated method to quantify urethral mobility in TPUS during cough, Valsalva maneuver, and PMC. This method, developed and validated in our prior study [5], uses optical flow‐based tracking of feature points within defined regions of interest (ROIs) to measure motion frame by frame throughout the full ultrasound cine loop. The only required operator intervention is the initial selection of the ROIs to be tracked. The tracking method is highly efficient, with analysis and production of results for each ultrasound cine loop requiring approximately the same time as viewing the loop itself. Although there is no limit on the number of regions that can be tracked, we selected three ROIs—proximal, mid, and distal—to correspond with the anatomical regions of the urethral support system described in the introduction. A representative example video illustrating this automated tracking is provided in the supporting material (Video S1). The method quantifies urethral displacement and rotation relative to a coordinate system fixed to the pubis, which corrects for probe‐pubis motion and enables consistent kinematic analysis across subjects. Detailed descriptions of the image‐processing and motion‐tracking algorithms are available in that publication [5]. Here, we briefly summarize the essential procedures and highlight specific methodological aspects relevant to the current study, which focuses on evaluating SUI in a clinical cohort.

### 2.1 Human Subjects

This prospective, case‐control study enrolled 21 adult women (age *>* 18 years): 10 continent controls and 11 with SUI. The inclusion criterion for SUI group was self‐reported symptom severity, defined as score *≥* 2 to item 17 of the Pelvic Floor Distress Inventory‐20 (PFDI‐20) [6]. Women were excluded if they had a history of pelvic organ prolapse, were undergoing cancer treatment, experienced chronic urinary tract infections, had received an organ transplant, sustained prior injuries or accidents involving the pelvic region, or had been pregnant or given birth within the preceding 3 months. Participant demographics are summarized in Table 1.

**TABLE 1:** Demographic and obstetric characteristics of study participants stratified by continence status.

Characteristic	Overall (*n* = 21)	Control (*n* = 10)	SUI (*n* = 11)	*p* value
Age (years)	46 ± 19 (20 − 76)	48 ± 21 (24 − 76)	45 ± 17 (20 − 72)	0.67^a^
BMI (kg*/*m^2^) Parity, *n* (%)	29 ± 7	27 ± 6	31 ± 8	0.14^a^ 0.21^b^
0	14 (66.7)	8 (80.0)	6 (54.5)	—
1–2^c^	6 (28.6)	2 (20.0)	4 (36.4)	—
*≥* 3	1 (4.7)	0 (0.0)	1 (9.1)	—

*Note:* Age and body‐mass index (BMI) are reported as mean ± standard deviation, whereas parity is reported as counts and percentages. For age, range is also reported.

^a^ Welch's two‐sample *t*‐test was used.

^b^ Mann‐Whitney U test compared the overall Parity distribution between groups.

^c^ Two participants with parity of 1 (1 Control, 1 SUI) had a cesarean section delivery; this variable was recorded but not analyzed statistically owing to the small counts.

### 2.2 Ultrasound Imaging

**FIGURE 2:**
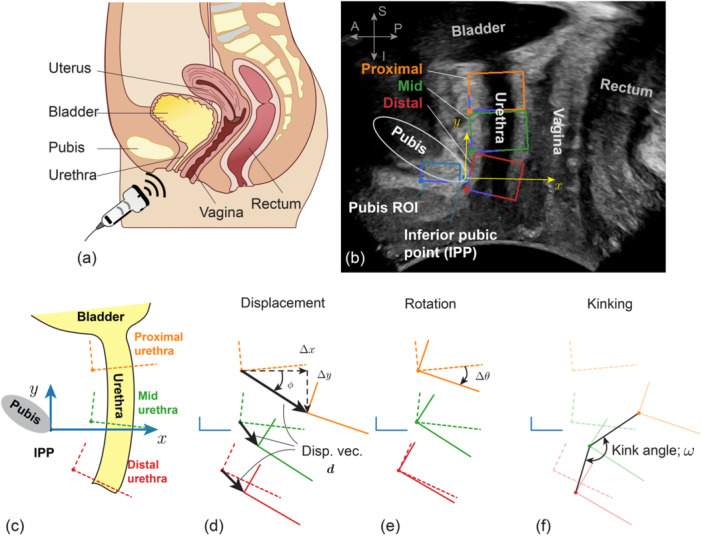
Anatomical and methodological setup for quantifying urethral mobility. (a) Schematic of sagittal view of a typical female pelvic floor showing key anatomical landmarks. (b) Example mid‐sagittal ultrasound image showing the bladder, vagina, rectum, pubis and the segmented urethral regions of interest (ROIs): proximal (orange), mid (green), and distal (red). The coordinate system for motion analysis is anchored at the IPP on the pubis. (c–e) Schematic representation of how displacement vectors and orientations are computed relative to the pelvic coordinate system. (f) Definition of the kink angle, *ω*.

TPUS was performed with participants in the lithotomy position using a GE HealthCare LOGIQ® E10 with a curvilinear C2‐9 probe (GE HealthCare, Wauwatosa, WI, USA). The transducer was positioned mid‐sagittally on the perineum with minimal pressure and allowed to move freely during maneuvers. A schematic illustration of the female pelvic floor and a representative mid‐sagittal TPUS B‐mode image are shown in Figure 2, with key urogenital structures labeled. The frame rate was set to the highest value allowed by depth and field of view (typically 30 Hz). The imaging plane and depth were adjusted to clearly visualize the bladder, urethra, and pubis and to accommodate the expected range of pelvic‐floor motion. Remaining scanning parameters (e.g., transmit frequency, mechanical index, and gain) were optimized per participant for optimal image quality.

During imaging, an experienced urogynecologist (J. O. D.) coached participants to carry out three repetitions of cough, Valsalva, and PMC, and ensured that maneuvers were performed correctly. For analysis, the trial that produced the greatest urethral excursion for each maneuver was selected.

### 2.3 Kinematic Metrics

The fundamental output generated by the automated tracking software is frame‐by‐frame positional information for each defined ROI. Specifically, it captures the position and orientation of each ROI's origin—defined as its bottom‐left corner, Figure 2b—relative to a fixed coordinate system anchored at the inferior pubic point (IPP) on the pubis. The positive *x*‐axis was defined as the straight line tangent to inferior margin of the pubis and pointed posteriorly, away from the pubis. The positive *y*‐axis was drawn perpendicular to *x*‐axis and pointed superiorly (Figure 2b,c).

From this basic positional data, numerous kinematic parameters can be computed. In this study we focus on metrics of clinical interest in the context of SUI. Specifically, we measured anterior‐posterior displacement (∆*x*), superior‐inferior displacement (∆*y*), and rotation of each segment (∆*θ*). From ∆*x* and ∆*y* we computed the displacement vector ***d*** = (∆*x*, ∆*y*), reporting its magnitude | ***d*** | and direction *φ*. We prioritized these two parameters in the main analysis, considering that they provide an integrated summary of urethral motion that is both biomechanically meaningful and clinically interpretable. Additionally, the “kink” angle (*ω*) between proximal‐to‐mid and mid‐to‐distal ROIs was quantified to evaluate urethral kinking. These metrics are shown schematically in Figure 2d–f. Finally, the differences in displacement magnitude between adjacent segments normalized by the distance between segments (proximal‐to‐mid and mid‐to‐distal) were also computed to quantify regional variations in urethral mobility.

### 2.4 Data Processing and Statistical Analysis

In our analysis, we first performed a 2 *×* 3 repeated‐measures ANOVA separately for each ROI, with group (control vs. SUI) as the between‐subject factor and event type (cough, Valsalva, PMC) as the within‐subject factor. We then conducted targeted pairwise comparisons using independent *t*‐tests (control vs. SUI at each event) and paired *t*‐tests (cough vs. Valsalva within each group), with Bonferroni corrections and significance set at *p* < 0.05. Each ROI was analyzed independently, rather than as a three‐way interaction (group × event × ROI), to simplify interpretation. All statistical analysis were confirmed by a statistician (S. D. N). Unless noted otherwise, mean and standard error of the mean (SEM) are reported throughout the study. Further details on data analyses are provided in Appendix A.

## 3 Results

**FIGURE 3:**
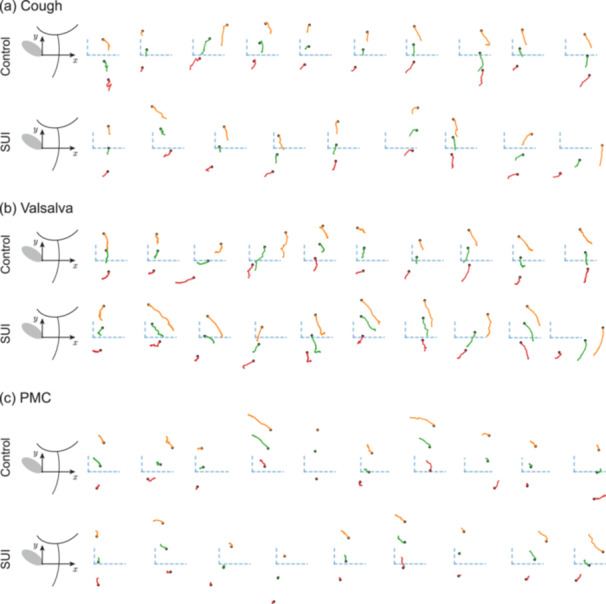
Each urethra displays unique patterns of movement. Trajectories of motion for the proximal (orange), mid (green) and distal (red) urethral segments are shown for all subjects in the control and SUI groups during (a) cough, (b) Valsalva maneuver, and (c) PMC. The order of subjects in each group is the same for each maneuver. Motion is plotted relative to the IPP‐attached coordinate system (dashed blue lines), with each trajectory showing the motion of the ROI's origin (defined as its bottom‐left corner) at time zero, marked by black circles. See Figure 2 for anatomical context.

Trajectories of each urethral segment during different maneuvers are shown for all subjects in Figure 3. For each subject and maneuver, the path traced by the ROI's origin is plotted relative to the IPP‐attached coordinate system. This visualization reveals substantial inter‐ subject variability in trajectory shape and excursion magnitude across maneuvers. These findings suggest that urethral mobility patterns are not only different between control and SUI subjects but each individual has its own unique signature.

**FIGURE 4:**
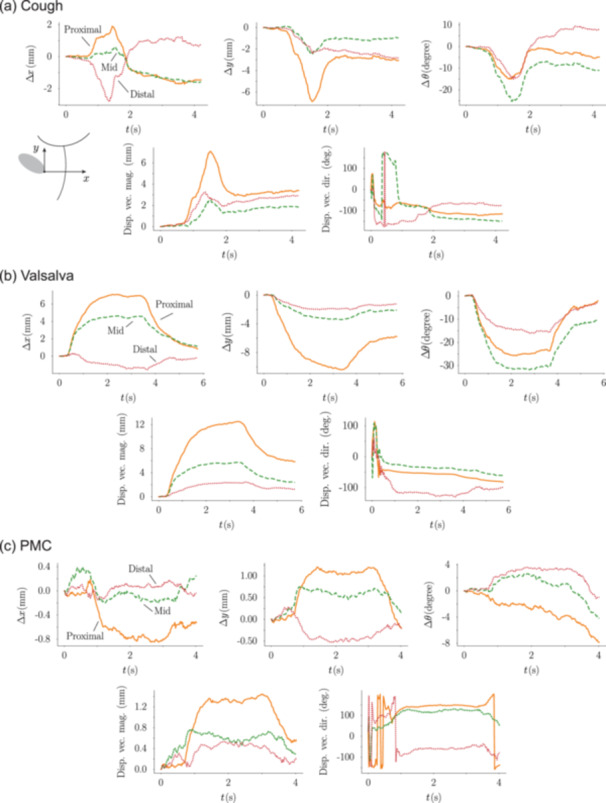
Example of urethral motion kinematics from a 42‐year‐old nulliparous SUI woman. Early fluctuations in displacement vector direction reflect low‐magnitude motion, where small positional changes produce large angular variability. (a) Cough. (b) Valsalva. (c) PMC.

Detailed motion quantification for a representative SUI subject during cough, Valsalva maneuver, and PMC is presented in Figure 4, illustrating the richness of the kinematic data that can be generated by this methodology. Careful examination of Figure 4 demonstrates how this dynamic information can provide clinical insights beyond those measurable from static images. For example, cough produces a rapid “spike‐and‐rebound” motion, whereas Valsalva results in slower, sustained displacement, indicative of maneuver‐specific viscoelastic responses. During cough, there are nearly equal yet opposite anterior‐posterior shifts along the urethra; the proximal segment moves posteriorly (∆*x* > 0), while the distal segment simultaneously shifts anteriorly (∆*x* < 0), both at comparable rates—this mirror‐symmetry is notably absent during Valsalva. Additionally, an anticipatory pelvic floor contraction during cough (seen as slight upward urethral motion preceding the main downward displacement) represents a subtle pre‐motion phenomenon entirely overlooked by traditional rest‐to‐ maximum assessments. Lastly, during PMC, the urethra initially moves posteriorly (∆*x* > 0) before reversing direction and drifting anteriorly (∆*x* < 0).

**FIGURE 5:**
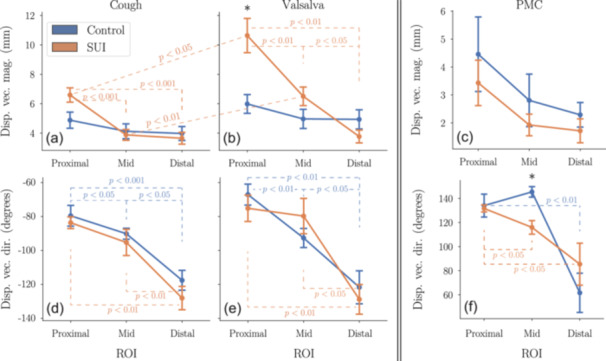
On average, the urethra moves more in the SUI group than in controls during stress events (cough and Valsalva), while during voluntary contraction, controls exhibit larger motion. Mean displacement magnitude (a–c) and direction (d–f) are shown for each urethral segment (proximal, mid, distal) during cough (a, d), Valsalva (b, e), and PMC (c, f). Error bars denote SEM. Statistically significant differences between groups (control vs. SUI) are marked with “*.” Significant differences between maneuvers (cough vs. Valsalva) and between urethral segments within the same group are indicated by dashed lines and associated *p*‐values. All *p*‐values were adjusted for multiple comparisons using the Bonferroni correction. See Appendix C of the supporting material for detailed statistical comparison and Figure 2 for anatomical definitions.

As previously mentioned, displacement vector magnitude and direction were prioritized in the main analysis due to their biomechanical and clinical relevance. Group‐level comparisons of these parameters are shown in Figure 5. Additional components are reported in Figure B1 of the supporting material for completeness. During stress maneuvers, SUI subjects exhibited larger displacement magnitudes than controls, with the most pronounced differences at the proximal urethra. This contrast was especially evident during Valsalva, where proximal displacement in the SUI group nearly doubled that of controls. Directional differences followed a similar pattern: SUI displacements were more inferiorly oriented, while controls showed relatively less downward deviation. During PMC controls demonstrated greater displacement magnitudes and more superior trajectories than SUI subjects, consistent with more effective pelvic floor engagement.

Displacement responses also varied by maneuver type. Within the SUI group, Valsalva consistently showed greater displacement magnitudes than cough, particularly at the proximal and mid urethra, where these differences reached statistical significance. This was not the case for controls, where no statistically significant differences were observed between cough and Valsalva.

Consistent with the variation in trajectory patterns observed in Figure 3, displacement vectors also exhibited substantial inter‐subject variability. Quantitative measures of this variability, including standard deviation, coefficient of variation, and range of values are presented in Table 2.

**TABLE 2:** Basic statistics of displacement vector, highlighting the inter‐subject variability in urethral mobility.

			Magnitude (mm)				Direction (degree)		
ROI	Group	Mean	SD	Range	CV (%)	Mean	SD	Range	CV (%)
**Cough**									
Proximal	Control	4.88	1.64	5.56	34	−79.55	18.39	43.38	23
	SUI	6.59	1.61	4.89	24	−83.65	10.34	28.88	12
Mid	Control	4.13	1.52	4.37	37	−90.14	8.55	23.81	9
	SUI	3.88	1.22	4.12	31	−95.26	25.55	87.78	27
Distal	Control	3.98	1.42	3.95	36	−117.56	17.49	49.94	15
	SUI	3.65	1.32	4.33	36	−128.03	22.75	72.73	18
**Valsalva**									
Proximal	Control	5.98	2.01	5.64	34	−67.08	18.56	60.64	28
	SUI	10.64	3.69	10.91	35	−75.16	24.52	61.48	33
Mid	Control	4.97	2.06	5.62	41	−92.64	16.79	50.94	18
	SUI	6.50	1.99	6.52	31	−79.73	32.86	87.93	41
Distal	Control	4.94	2.06	5.95	42	−121.68	30.73	97.41	25
	SUI	3.77	1.38	4.63	37	−128.79	26.19	89.40	20
**PMC**									
Proximal	Control	4.46	4.01	11.95	90	134.06	25.17	64.58	19
	SUI	3.43	2.58	9.39	75	131.66	7.66	22.04	6
Mid	Control	2.81	2.82	9.19	101	145.42	10.69	29.77	7
	SUI	1.93	1.22	3.38	64	115.97	16.90	52.99	15
Distal	Control	2.29	1.32	3.62	58	61.67	43.04	113.79	70
	SUI	1.72	1.35	3.77	79	85.45	49.28	161.15	58

*Note:* Range: Maximum value ‐ minimum value.

Abbreviations: CV, coefficient of variance (SD/mean); SD, standard deviation.

**FIGURE 6:**
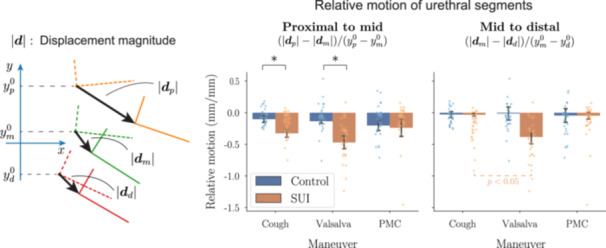
Urethral displacement is more spatially non‐uniform in the SUI group, particularly during stress maneuvers. Relative motion of urethra (the change in displacement magnitude by the distance between segments in the *y*‐direction) is shown for proximal‐to‐mid and mid‐to‐distal segments, for each group during cough, Valsalva maneuver, and PMC. Negative values indicate greater motion in the more proximal segment. Error bars are SEM. Statistically significant differences between groups (controls vs. SUI) are marked with “*”, while significant differences between maneuvers (cough vs. Valsalva) within the same group are indicated by dashed lines and associated *p*‐values. All *p*‐values were adjusted for multiple comparisons using the Bonferroni correction. See Figure 2 for anatomical context. The details of statistical comparisons are provided in Appendix C of the supporting materials.

Relative mobility of urethral segments—quantifying how much the mid urethra moves relative to the proximal segment, and how much the distal segment moves relative to the mid segment during each maneuver—is shown in Figure 6. Consistent with Figures 3 and 5, control subjects typically exhibit uniform motion along the urethra. In contrast, SUI subjects display non‐uniform motion, with the proximal urethra demonstrating significantly greater movement compared to distal segents during stress events.

**FIGURE 7:**
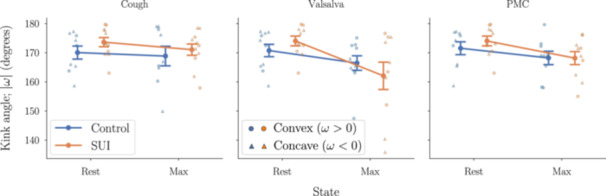
There is no significant difference in kink angle (*ω*) between SUI and control groups at either rest or maximum excursion. While the urethra tends to kink more during Valsalva in the SUI group, this difference is not statistically significant. Discrete overlaid points represent individual subjects; circles indicate convex kinking (*ω* > 0) and triangles indicate concave kinking (*ω* < 0). Bold markers with associated error bars denote group means ± SEM. See Figure 2 for kinking definition.

To complement this analysis, we also examined the degree to which the urethra bends; clinically referred to as “kinking,” which is often presumed to reflect abnormal mobility or support. Kink angle—defined as the angle formed between adjacent urethral segments (Figure 2f)—at both rest and at maximum excursion is shown in Figure 7 for each maneuver. While there was a trend toward greater kinking during Valsalva in the SUI group, this difference did not reach statistical significance. Detailed values and statistical comparisons for the kinematic metrics considered in this study are provided in the supporting material (Appendix C).

**FIGURE 8:**
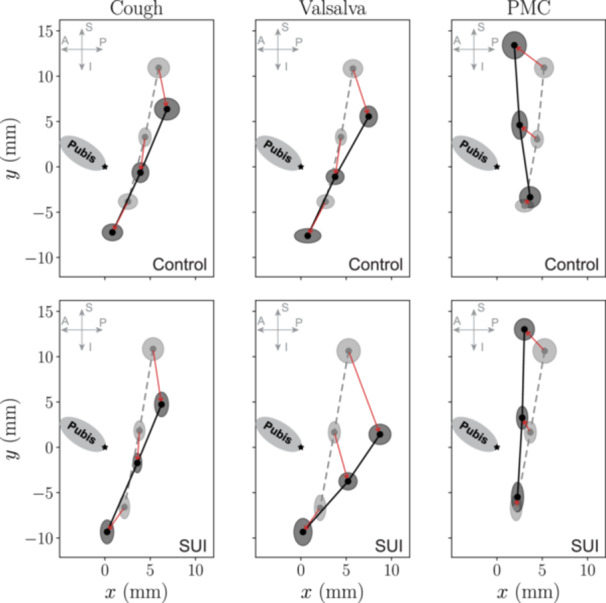
Urethral displacement patterns at maximum excursion differ between control (top row) and SUI (bottom row) groups. For each maneuver (cough, Valsalva, PMC), the mean positions of the proximal, mid, and distal urethral ROIs origins at rest (light gray outlines with dashed lines) and at maximum excursion (dark gray outlines with solid lines) are shown relative to the IPP‐attached coordinate system, see Figure 2. Ellipses indicate the SEM in both *x* and *y* directions across subjects. The red arrows show displacement vector. During cough and Valsalva, SUI subjects show greater and more posterior displacement, especially at the proximal urethra, while control subjects exhibit smaller excursion with less posterior bias. In contrast, PMC responses in controls are superiorly directed across urethral segments, whereas SUI subjects show minimal or no displacement.

The mean positions of each urethral segment at rest and at maximum excursion during cough, Valsalva, and PMC are shown in Figure 8, plotted in the IPP‐attached coordinate system. SEM is shown as oval representing the variation in *x* and *y* directions. These plots provide a spatial overview of group‐averaged urethral motion trajectories.

## 4 Discussion

How and why the urethra loses support when IAP increases is poorly understood, contributing to the persistence of SUI as a common pelvic floor disorder. Although urethral hypermobility's role in SUI is well‐established [1], clinical evaluation still largely relies on maximal excursion measurements [7, 8, 9], failing to capture the complexity of dynamic urethral motion in ways that could be linked directly to anatomical supports. This requires detailed kinematics data from specific urethral regions. Although such analyses have previously been undertaken [3, 4], their cumbersome nature has hindered clinical adoption. Here, we demonstrate a scalable, automated ultrasound method capable of quantifying detailed urethral kinematics throughout the full ultrasound cine loop of any format, generating rich, clinically relevant data with high efficiency and minimal operator intervention.

Our inter‐group comparisons show that, on average, women with SUI experience greater and more inferiorly directed urethral displacements during stress events, particularly at the proximal urethra. This pronounced bladder neck descent strongly supports the classical concept of urethral hypermobility, highlighting a loss of the normal supportive hammock provided by the levator ani muscles, anterior vaginal wall and endopelvic fascia [10, 11]. In a well‐supported pelvic floor, increases in IAP are transmitted to a stable sub‐urethral layer that braces and compresses the urethra closed. During voluntary PMC, the pattern of motion was reversed. Continent women demonstrated larger superior displacement across all urethral segments, reflecting a coordinated lifting by the pelvic floor muscles. Women with SUI, on the other hand, showed reduced urethral elevation during PMC, indicating impaired voluntary pelvic‐floor muscle activation, consistent with clinical observations of weakened or uncoordinated musculature in SUI [12]. However, substantial variability and overlap between groups indicate the need for more nuanced analysis.

Our data show that in women with SUI, Valsalva produces larger and more posterior inferior urethral excursions compared to cough, even though coughing generally generates higher peak IAPs [13]. Clinically, this aligns with the scenario where patients remain continent during brief coughs yet leak during prolonged straining. Valsalva exposes viscoelastic tissues around the bladder neck to prolonged mechanical load, allowing greater tissue deformation and displacement (“creep”), whereas coughing involves a rapid pressure spike often mitigated by a protective, preprogrammed PMC (“preflex”). Clinically, these distinct displacement patterns highlight different potential failure modes in SUI: leakage predominantly with Valsalva may indicate primarily connective tissue laxity, whereas leakage during cough suggests compromised connective tissue coupled with impaired neuromuscular activation.

We also observed non‐uniform motion along the urethra, characterized by greater mobility proximally and relatively restricted motion distally. By contrast, continent controls exhibited uniform and coordinated urethral displacement across all segments. This decay in displacement from proximal to distal urethral segments is most pronounced during Valsalva and is consistent with prior observations of disproportionate mobility, particularly at the urethrovesical junction [7].

Interestingly, our analysis did not identify prominent urethral kinking in the SUI group, despite its frequent mention as a characteristic of urethral hypermobility in SUI [14]. Both SUI and control subjects demonstrated a mix of convex and concave kinking patterns, with considerable inter‐subject variability. The kink angle may therefore lack the sensitivity or specificity to evaluate SUI, especially when compared to the relative mobility of urethral segments. It is important to note that our cohort did not include patients with large cystoceles, a factor that might account for the observed lack of kinking.

While comparisons of women with SUI to continent controls in this study align with established literature, the most promising aspect of our methodology lies in its ability to provide detailed information on motion in terms of magnitude, direction and rotation for each individual woman. The substantial inter‐subject variability we observed underscores the inherent heterogeneity of urethral support mechanisms. Identifying and quantifying this variability reveals how distinct urethral mobility patterns may indicate fundamentally different pathophysiological mechanisms, paving the way for personalized phenotyping and individualized treatment strategies.

**FIGURE 9:**
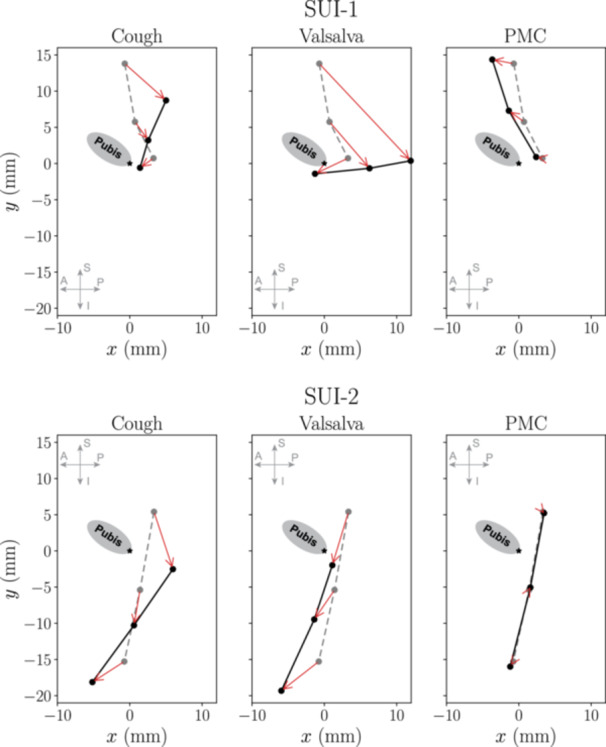
Comparison of urethral displacement patterns between two SUI patients suggesting distinct failure modes. In SUI‐1 (top row), the urethra initially occupies a superior resting position and exhibits substantial posterior‐inferior displacement, particularly during Valsalva, consistent with structural laxity and hypermobility. Additionally, SUI‐1 achieves effective urethral lift during voluntary pelvic floor contraction. In contrast, SUI‐2 (bottom row) shows a more inferior‐posterior initial urethral position, reduced displacement during stress maneuvers, and negligible urethral lift during PMC, suggesting compromised levator ani. These distinct kinematic patterns highlight the potential for mechanistic phenotyping of SUI to guide personalized treatment strategies.

An example of such phenotyping is shown in Figure 9, where urethral displacement patterns of two SUI patients are compared. In SUI 1, the urethra initially occupied a more superior resting position relative to the pubis and displayed pronounced mobility during stress events, particularly at the proximal segment. Despite this marked hypermobility, the patient achieved effective upward lift during voluntary PMC, suggesting intact levator ani muscle function. These characteristics align closely with structural laxity of endopelvic fascia. In contrast, in SUI 2, the urethra was initially positioned more inferiorly and posteriorly and exhibited reduced mobility during stress events in an anterior‐inferior direction, with minimal variation along the urethral length. This suggests that fascial laxity might not be the predominant failure mechanism. Additionally, voluntary PMC resulted in essentially no effective urethral lift, producing negligible displacement, pointing to impaired levator ani muscle function. While these two examples illustrate how individual kinematic profiles can provide insights into distinct SUI phenotypes, these findings remain exploratory and require further validation. These observations should be regarded as hypothesis‐generating rather than definitive mechanistic conclusions. Future studies with larger and more demographically diverse cohorts, inclusion of additional pelvic floor conditions such as prolapse, and independent ground‐truth measures for different SUI mechanisms will be needed to confirm these observations.

### 4.1 Clinical Implications

From a clinical standpoint, this study shows that dynamic ultrasound of the urethra can add information beyond a single maximum excursion measure used in current practice. Our findings underscore that urethral motion during stress events is not uniform along the urethra. In a well‐supported pelvic floor, the proximal urethra is braced by connective tissue and the levator ani muscles, while the distal urethra is relatively fixed at the perineal membrane. In some SUI cases, our analysis revealed exaggerated inferior‐posterior displacement at the proximal urethra, suggesting loss of support. In other cases, reduced elevation during voluntary pelvic floor contraction points to impaired levator ani muscle function. Together, these patterns indicate that stress leakage can arise from different underlying mechanisms even when symptoms are similar. If validated in larger and more diverse cohorts, this approach could help clinicians move beyond describing hypermobility in general terms toward identifying the dominant failure mechanism in each patient. Such individualized phenotyping could guide treatment selection, for example favoring pelvic floor muscle training when activation is deficient, or surgical reinforcement when connective tissue support is compromised. Compared with existing bedside tools, this method provides more objective and comprehensive information. The Q‐tip test is difficult to perform reliably: repositioning the goniometer during Valsalva introduces error at the point of origin, and the test cannot be used during cough. Similarly, grading urethral mobility on exam is subjective and varies between examiners. By contrast, our automated ultrasound approach provides a re‐producible, maneuver‐independent assessment of urethral motion. Importantly, because the analysis is automated, requires only initial region selection, and runs in about the time it takes to view the cine loop, it can be incorporated into routine imaging to guide therapy. The tracking method itself requires no specialized training other than familiarity with pelvic floor anatomy. It does not depend on training datasets or advanced machine‐learning infrastructure and is based on transparent and lightweight computer vision algorithms. Combined with the growing availability of inexpensive handheld ultrasound devices, this lowers the barrier to adoption and makes it feasible for every urodynamic clinic to integrate dynamic urethral imaging into routine practice.

Lastly, the rich, quantitative kinematic data that can be generated with this method could readily serve as structured input for predictive models. For example, future machine‐learning models could identify unique patterns, classify women with SUI into different failure modes, predict surgical outcomes, or refine risk stratification in women with borderline continence.

### 4.2 Limitations

This is a feasibility study and has several limitations. The modest number of participants restricts the statistical power and the generalizability of our findings. Although the continent and SUI groups were reasonably matched and did not differ significantly in age, BMI, or parity (Table 1), the study was underpowered to explore subgroup effects or interactions across these factors. For the same reason of limited sample size, we focused on ROI‐specific analyses with correction for multiple comparisons, an approach that balances interpretability with rigor in a small exploratory dataset. We acknowledge that more integrative statistical models (e.g., mixed‐effects approaches considering group × event × ROI interactions) could in larger samples yield further insights. Another factor that might affect reproducibility is that all ultrasound scanning was performed by a single urogynecologist. We also excluded women with pelvic organ prolapse in this feasibility study, restricting applicability to the broader population of patients with pelvic floor disorders. Lastly, while our discussion of potential SUI mechanisms is supported by prior observations, these interpretations remain speculative, requiring further experimental validation.

## 5 Conclusions

Our study demonstrates that the proposed automated, ultrasound‐based approach recapitulates known urethral mobility patterns. By capturing full dynamic motion—rather than merely rest‐to‐maximum excursions—it provides biomechanical insights otherwise lost. The substantial variability and overlap observed between groups highlight the need for more nuanced analysis. The proposed methodology is highly efficient, requires minimal operator intervention, analyzes data in about the same time it takes to view the ultrasound cine loops.

## Author Contributions

**Kourosh Kalayeh:** conceptualization, methodology, software development, imaging and data acquisition, analysis and interpretation of the results, drafting and revising the manuscript, approval of the manuscript. **J. Brian Fowlkes:** conceptualization, methodology, software development, imaging and data acquisition, analysis and interpretation of the results, revising the manuscript, approval of the manuscript. **Stephanie Daignault‐Newton:** data and statistical analysis, revising the manuscript, approval of the manuscript. **Payton Schmidt:** Imaging and data acquisition, revising the manuscript, approval of the manuscript. **James A. Ashton‐Miller:** conceptualization, methodology, analysis and interpretation of the results, revising the manuscript, approval of the manuscript. **John O. DeLancey:** conceptualization, methodology, imaging and data acquisition, analysis and interpretation of the results, revising the manuscript, approval of the manuscript.

## Ethics Statement

This study was approved by the Institutional Review Board (IRB) at the University of Michigan, under approval number HUM00181787. The study complied with the Health Insurance Portability and Accountability Act (HIPAA).

## Consent

Informed consent was obtained from all individual participants included in the study.

## Conflicts of Interest

The authors declare no conflicts of interest.

## Supporting information

jnls‐nau_2025‐r2‐sui‐kalayeh_etal‐urethral_motion_tracking_eg‐valsalva‐img.

## Figures and Tables

**FIGURE B1:**
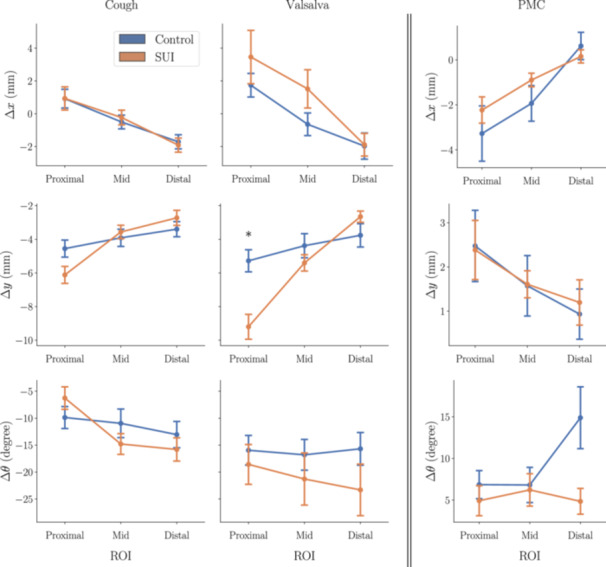
Component‐wise urethral displacement and segmental rotation at maximum excursion. Mean and SEM values are shown for anterior‐posterior displacement (∆*x*), superiorinferior displacement (∆*y*), and segmental rotation (∆*θ*) across the proximal, mid, and distal urethral ROIs during cough, Valsalva maneuver, and PMC. Data are grouped by continence status (control vs. SUI). This figure expands on Figure 5 by decomposing total displacement into directional components and rotation. Statistical significant differences between SUI and control groups is marked by “*.” See Figure 2 for anatomical context.

## Data Availability

The data that support the findings of this study are available from the corresponding author upon reasonable request.

## Appendix A Open‐Source Software Packages Used for Data Analysis

Data analyses were done with open‐source Python programming language, specifically NumPy [15], SciPy [16], Pandas [17], Matplotlib [18], and seaborn [19] libraries. Pydicom [20] library was used to access and read the DICOM (Digital Imaging and Communications in Medicine) files. The following version of these libraries were used.

1. Python 3.12.9
2. matplotlib ==3.9.1
3. numpy = =1.26.4 4 pandas = =2.2.2
4. pydicom = =2.4.4
5. scipy ==1.14.0

## Appendix C Detailed Quantitative Comparison of Key Metrics

Detailed quantitative comparisons of key urethral kinematic metrics across urethral ROIs (proximal, mid, distal) and maneuvers (cough, Valsalva, PMC) for control and SUI groups are presented in this section. Values are reported as mean ± SEM. Statistical significance for differences between groups (control vs. SUI) and within‐group comparisons (Valsalva vs. cough, proximal vs. mid/distal segments) are indicated with adjusted *p*‐values (Bonferroni correction), denoted by letters indicating comparison groups (e.g., 0.015 V means *p* = 0.015 vs. Valsalva; *<* 0.001 *M* means *p* < 0.001 vs. mid‐segment). Effect sizes (Cohen's d) are also reported. No significance differences are denoted by dashed lines (—).

See Table C1

**TABLE C1:** Displacement magnitude (*|d* | ).

ROI	Group	Mean *± *SEM (mm)	Statistically significant (*p*‐value^a^)	Effect size (Cohen's d)
**C: Cough**				
P: Proximal	Control	4.88 ± 0.55	—	0.8 V, 0.7 M, 0.9D
	SUI	6.59 ± 0.49	0.015 V, *<* 0.001 M, *<* 0.001D	1.1 V, 1.9 M, 1.7D
	Control versus SUI		—	1.1
M: Mid	Control	4.13 ± 0.51	—	0.9 V, 0.7 P, 0.2D
	SUI	3.88 ± 0.37	0.006 V, *<* 0.001 P	1.3 V, 1.9 P, 0.4D
	Control versus SUI		—	0.2
D: Distal	Control	3.98 ± 0.47	—	1.1 V, 0.9 P, 0.2 M
	SUI	3.65 ± 0.40	*<* 0.001 P	0.0 V, 1.7 P, 0.4 M
	Control versus SUI		—	0.2
**V: Valsalva**				
P: Proximal	Control	5.98 ± 0.64	0.008 M	0.8 C, 1.3 M, 0.5D
	SUI	10.64 ± 1.17	0.015 C, 0.003 M, 0.001D	1.1 C, 1.5 M, 1.7D
	Control versus SUI		0.008	1.6
M: Mid	Control	4.97 ± 0.65	0.008 P	0.9 C, 1.3 P, 0.0D
	SUI	6.50 ± 0.63	0.006 C, 0.003 P, 0.023D	1.3 C, 1.5 P, 1.1D
	Control versus SUI		—	0.8
D: Distal	Control	4.94 ± 0.65	—	1.1 C, 0.5 P, 0.0 M
	SUI	3.77 ± 0.44	0.001 P, 0.023 M	0.0 C, 1.7 P, 1.1 M
	Control versus SUI		—	0.7
**K: PMC**				
P: Proximal	Control	4.46 ± 1.34	—	0.9 M, 0.7D
	SUI	3.43 ± 0.81	—	0.8 M, 0.8D
	Control versus SUI		—	0.3
M: Mid	Control	2.81 ± 0.94	—	0.9 P, 0.2D
	SUI	1.93 ± 0.39	—	0.8 P, 0.2D
	Control versus SUI		—	0.4
D: Distal	Control	2.29 ± 0.44	—	0.7 P, 0.2 M
	SUI	1.72 ± 0.43	—	0.8 P, 0.2 M
	Control versus SUI		—	0.4

^a^ Bonferroni adjusted for multiple comparisons. Independent *t*‐test for between SUI and control groups and paired *t*‐test for within group comparisons.

See Table C2

**TABLE C2:** Displacement vector direction (*ϕ*).

ROI	Group	Mean *± *SEM (mm)	Statistically significant (*p*‐value^a^)	Effect size (Cohen's d)
**C: Cough**				
P: Proximal	Control SUI	*−*79.55 ± 6.13 *−*83.65 ± 3.45	0.010 M, *<* 0.001D 0.003D	0.8 V, 1.8 M, 2.4D 0.9 V, 0.9 M, 1.7D
	Control versus SUI		—	0.3
M: Mid	Control SUI	*−*90.14 ± 3.23 *−*95.26 ± 7.70	0.010 P, 0.011D 0.006D	0.2 V, 1.8 P, 1.7D 0.8 V, 0.9 P, 1.3D
	Control versus SUI		—	0.2
D: Distal	Control	*−*117.56 ± 5.83	*<* 0.001 P, 0.011 M	0.1 V, 2.4 P, 1.7 M
	SUI	*−*128.03 ± 6.86	0.003 P, 0.006 M	0.3 V, 1.7 P, 1.3 M
	Control versus SUI		—	0.5
**V: Valsalva**				
P: Proximal	Control SUI	*−*67.08 ± 6.19 *−*75.16 ± 7.75	0.008 M, 0.007D 0.004D	0.8 C, 1.4 M, 1.5D 0.9 C, 0.3 M, 1.6D
	Control versus SUI		—	0.4
M: Mid	Control	*−*92.64 ± 5.60	0.008 P, 0.028D	0.2 C, 1.4 P, 1.1D
	SUI	*−*79.73 ± 10.39	0.016D	0.8 C, 0.3 P, 1.3D
	Control versus SUI		—	0.5
D: Distal	Control	*−*121.68 ± 9.72	0.007 P, 0.028 M	0.1 C, 1.5 P, 1.1 M
	SUI	*−*128.79 ± 8.73	0.004 P, 0.016 M	0.3 C, 1.6 P, 1.3 M
	Control versus SUI		—	0.2
**K: PMC**				
P: Proximal	Control	134.06 ± 9.51	0.009D	0.3 M, 2.2D
	SUI	131.66 ± 2.89	0.044 M, 0.022D	1.3 M, 1.8D
	Control versus SUI		—	0.1
M: Mid	Control	145.42 ± 4.36	—	0.3 P, 1.3D
	SUI	115.97 ± 5.63	0.044 P	1.3 P, 0.6D
	Control versus SUI		0.007	2.0
D: Distal	Control	61.67 ± 16.27	0.009 P	2.2 P, 1.3 M
	SUI	85.45 ± 17.42	0.022 P	1.8 P, 0.6 M

*Note:* Control versus SUI0.5.

^a^ Bonferroni adjusted for multiple comparisons. Independent *t*‐test for between SUI and control groups and paired *t*‐test for within group comparisons.

See Table C3

**TABLE C3:** Anterior‐posterior displacement (∆*x*).

ROI	Group	Mean *± *SEM (mm)	Statistically significant (*p*‐value^a^)	Effect size (Cohen's d)
**C: Cough**				
P: Proximal	Control	0.92 ± 0.57	0.006 M, 0.010D	0.3 V, 1.5 M, 1.4D
	SUI	0.93 ± 0.71	0.010D	0.6 V, 0.8 M, 1.2D
	Control versus SUI		—	0.0
M: Mid	Control SUI	*−*0.51 ± 0.42 *−*0.23 ± 0.45	0.006 P, 0.033D 0.004D	0.1 V, 1.5 P, 1.1D 0.5 V, 0.8 P, 1.3D
	Control versus SUI		—	0.2
D: Distal	Control SUI	*−*1.71 ± 0.43 *−*1.92 ± 0.43	0.010 P, 0.033 M 0.010 P, 0.004 M	0.2 V, 1.4 P, 1.1 M 0.1 V, 1.2 P, 1.3 M
	Control versus SUI		—	0.1
**V: Valsalva**				
P: Proximal	Control	1.74 ± 0.72	0.001 M, *<* 0.001D	0.3 C, 1.7 M, 2.0D
	SUI	3.46 ± 1.63	0.041D	0.6 C, 0.8 M, 1.0D
	Control versus SUI		—	0.4
M: Mid	Control SUI	*−*0.64 ± 0.69 1.51 ± 1.17	0.001 P, 0.031D 0.048D	0.1 C, 1.7 P, 1.0D 0.5 C, 0.8 P, 0.9D
	Control versus SUI		—	0.7
D: Distal	Control SUI	*−*1.97 ± 0.80 *−*1.89 ± 0.69	*<* 0.001 P, 0.031 M 0.041 P, 0.048 M	0.2 C, 2.0 P, 1.0 M0.1 C, 1.0 P, 0.9 M
	Control versus SUI		—	0.0
**K: PMC**				
P: Proximal	Control	*−*3.28 ± 1.23	0.012D	0.7 M, 1.3D
	SUI	*−*2.23 ± 0.58	0.027 M, 0.038D	1.1 M, 1.0D
	Control versus SUI		—	0.4
M: Mid	Control	*−*1.94 ± 0.79	0.008D	0.7 P, 1.4D
	SUI	*−*0.90 ± 0.30	0.027 P	1.1 P, 0.7D
	Control versus SUI		—	0.6
D: Distal	Control	0.62 ± 0.61	0.012 P, 0.008 M	1.3 P, 1.4 M
	SUI	0.15 ± 0.29	0.038 P	1.0 P, 0.7 M
	Control versus SUI		—	0.3

^a^ Bonferroni adjusted for multiple comparisons. Independent *t*‐test for between SUI and control groups and paired *t*‐test for within group comparisons.

See Table C4

**TABLE C4:** Superior‐inferior displacement (∆*y*).

ROI	Group	Mean *± *SEM (mm)	Statistically significant (*p*‐value^a^)	Effect size (Cohen's d)
**C: Cough**				
P: Proximal	Control	*−*4.56 ± 0.50	—	0.4 V, 0.5 M, 0.9D
	SUI	*−*6.12 ± 0.50	0.020 V, *<* 0.001 M, *<* 0.001D	1.0 V, 2.1 M, 2.2D
	Control versus SUI		—	1.0
M: Mid	Control	*−*3.91 ± 0.51	—	0.1 V, 0.5 P, 0.6D
	SUI	*−*3.57 ± 0.40	0.016 V, *<* 0.001 P, 0.007D	1.1 V, 2.1 P, 1.2D
	Control versus SUI		—	0.2
D: Distal	Control	*−*3.40 ± 0.45	—	0.3 V, 0.9 P, 0.6 M
	SUI	*−*2.73 ± 0.45	*<* 0.001 P, 0.007 M	0.0 V, 2.2 P, 1.2 M
	Control versus SUI		—	0.5
**V: Valsalva**				
P: Proximal	Control	*−*5.28 ± 0.66	0.037D	0.4 C, 0.9 M, 1.0D
	SUI	*−*9.20 ± 0.74	0.020 C, *<* 0.001 M, *<* 0.001D	1.0 C, 1.9 M, 2.5D
	Control versus SUI		0.003	1.8
M: Mid	Control	*−*4.38 ± 0.72	—	0.1 C, 0.9 P, 0.4D
	SUI	*−*5.41 ± 0.48	0.016 C, *<* 0.001 P, 0.001D	1.1 C, 1.9 P, 1.7D
	Control versus SUI		—	0.5
D: Distal	Control	*−*3.76 ± 0.70	0.037 P	0.3 C, 1.0 P, 0.4 M
	SUI	*−*2.66 ± 0.33	*<* 0.001 P, 0.001 M	0.0 C, 2.5 P, 1.7 M
	Control versus SUI		—	0.6
**K: PMC**				
P: Proximal	Control	2.47 ± 0.81	—	0.6 M, 0.6D
	SUI	2.38 ± 0.67	—	0.5 M, 0.6D
	Control versus SUI		—	0.0
M: Mid	Control	1.57 ± 0.68	—	0.6 P, 0.4D
	SUI	1.61 ± 0.31	—	0.5 P, 0.3D
	Control versus SUI		—	0.0
D: Distal	Control	0.93 ± 0.57	—	0.6 P, 0.4 M
	SUI	1.19 ± 0.51	—	0.6 P, 0.3 M
	Control versus SUI		—	0.2

^a^ Bonferroni adjusted for multiple comparisons. Independent *t*‐test for between SUI and control groups and paired *t*‐test for within group comparisons.

See Table C5.

**TABLE C5:** Segmental rotation (∆*θ*).

ROI	Group	Mean *± *SEM (mm)	Statistically significant (*p*‐value^a^)	Effect size (Cohen's d)
**C: Cough**				
P: Proximal	Control	*−*9.87 ± 2.04	—	0.6 V, 0.2 M, 0.5D
	SUI	*−*6.26 ± 2.09	0.018 V, *<* 0.001 M, 0.008D	1.0 V, 1.7 M, 1.2D
	Control versus SUI		—	0.5
M: Mid	Control	*−*10.96 ± 2.65	—	0.7 V, 0.2 P, 0.5D
	SUI	*−*14.79 ± 1.92	*<* 0.001 P	0.5 V, 1.7 P, 0.2D
	Control versus SUI		—	0.5
D: Distal	Control	*−*13.05 ± 2.44	—	0.1 V, 0.5 P, 0.5 M
	SUI	*−*15.80 ± 2.16	0.008 P	0.6 V, 1.2 P, 0.2 M
	Control versus SUI		—	—
P: Proximal	Control	*−*15.96 ± 2.75	—	0.6 C, 0.1 M, 0.0D
	SUI	*−*18.58 ± 3.70	0.018 C	1.0 C, 0.3 M, 0.5D
	Control versus SUI		—	0.3
M: Mid	Control	*−*16.80 ± 2.85	—	0.7 C, 0.1 P, 0.1D
	SUI	*−*21.29 ± 4.84	—	0.5 C, 0.3 P, 0.2D
	Control versus SUI		—	0.4
D: Distal	Control	*−*15.69 ± 3.03	—	0.1 C, 0.0 P, 0.1 M
	SUI	*−*23.31 ± 4.78	—	0.6 C, 0.5 P, 0.2 M
	Control versus SUI		—	0.6
**K: PMC**				
P: Proximal	Control	6.85 ± 1.68	—	0.0 M, 0.7D
	SUI	4.92 ± 1.80	—	0.2 M, 0.0D
M: Mid	Control	6.81 ± 2.12	—	0.0 P, 0.8D
	SUI	6.22 ± 1.94	—	0.2 P, 0.3D
D: Distal	Control	14.88 ± 3.71	—	0.7 P, 0.8 M
	SUI	4.85 ± 1.55	—	0.0 P, 0.3 M

*Note:* Control versus SUI1.2.

^a^ Bonferroni adjusted for multiple comparisons. Independent *t*‐test for between SUI and control groups and paired *t*‐test for within group comparisons.
